# The dark septate endophyte *Phialocephala sphaeroides* confers growth fitness benefits and mitigates pathogenic effects of *Heterobasidion* on Norway spruce

**DOI:** 10.1093/treephys/tpab147

**Published:** 2021-11-15

**Authors:** Zilan Wen, Eeva Terhonen, Fred O Asiegbu

**Affiliations:** Faculty of Agriculture and Forestry, University of Helsinki, Helsinki 00790, Finland; Natural Resources Institute Finland (Luke), Helsinki 00790, Finland; Faculty of Agriculture and Forestry, University of Helsinki, Helsinki 00790, Finland

**Keywords:** fungi endophyte, growth promotion, *Heterobasidion* infection, plant defense response, transcriptome

## Abstract

Forest trees frequently interact with a diverse range of microorganisms including dark septate endophytes (DSEs) and fungal pathogens. Plant defense responses to either individual pathogens or endophytes have been widely studied, but very little is known on the effect of coinfection on host defenses. To study the impact of coinfection or tripartite interaction on plant growth and host defenses, Norway spruce (*Picea abies* (L.) Karst) seedlings were inoculated with a DSE *Phialocephala sphaeroides* or with a root pathogen *Heterobasidion parviporum* Niemela & Korhonen or coinfected with both fungi. The results showed that the DSE promoted the root growth of spruce seedlings. Control seedlings without any inoculum were subjected to sequencing and used as a baseline for identification of differentially expressed genes (DEGs). RNA-seq analysis of seedlings inoculated with *P. sphaeroides*, infected with *H. parviporum* or coinfected with both fungi resulted in a total of 5269 DEGs. The majority of DEGs were found in *P. sphaeroides*-inoculated seedlings. Lignin biosynthesis pathways were generally activated during fungal infections. The pattern was distinct with endophyte inoculation. The majority of the genes in the flavonoid biosynthesis pathway were generally suppressed during fungal infections. A specific transcriptional response to *P. sphaeroides* inoculation was the increased transcripts of genes involved in jasmonic acid biosynthesis, mitogen-activated protein kinases signaling pathway, plant hormone signal transduction and calcium-mediated signaling. This may have potentially contributed to promoting the root growth of seedlings. Although the coinfection suppressed the induction of numerous genes, no negative effect on the growth of the spruce seedlings occurred. We conclude that the subsequent *H. parviporum* infection triggered reprogramming of host metabolism. Conversely, the endophyte (*P. sphaeroides*), on the other hand, counteracted the negative effects of *H. parviporum* on the growth of the spruce seedlings.

## Introduction

*Heterobasidion parviporum* Niemelä & Korhonen naturally infects Norway spruce (*Picea abies* (L.) Karst) and causes root and stem rot. This necrotrophic pathogen occurs in the northern hemisphere (Garbelotto and Gonthier 2013). The pathogen is especially common in southern Finland where the disease is spreading gradually toward the north probably due in part to climate change (Venäläinen et al. 2020). Primary infection occurs in freshly cut stumps exposed to aerial basidiospores. The spores that adhere to the surface of the stumps form invasive hyphae, which subsequently spread to neighboring healthy trees via root contact (Asiegbu et al. 2005). Although new *H. parviporum* infections can be prevented by spraying the chemical (urea) or *Phlebiopsis gigantea* (Fr.) Jül on the freshly cut stumps, the development of other alternative approaches to the current control methods deserve to be explored. Dark septate endophytes (DSEs) with melanized and septate hyphae such as *Phialocephala* species are the most abundant fungi that colonize roots of Norway spruce (Grünig et al. 2006, Terhonen et al. 2014). Particularly, the finding that *Phialocephala sphaeroides* promoted the root growth of spruce seedlings and inhibited the growth of *H. parviporum* (Terhonen et al. 2016) suggests that this DSE has the potential to be explored in disease management. Understanding how *P. sphaeroides* mediates root growth at the molecular level formed one of the objectives of this study.

The abundance and broad host range of DSEs indicate that they have an important function in nature. They do not have a preference for host species and have been isolated from over 600 plant species (Jumpponen and Trappe 1998). Their effects on plant growth vary from negative to neutral (Tellenbach et al. 2011) and to positive (Jumpponen and Trappe 1998, Alberton et al. 2010), depending on the host, fungal genotype and environmental conditions (Fernando and Currah 1996, Jumpponen 2001, Reininger et al. 2012). Beneficial DSEs, colonizing root tissues intracellularly and intercellularly, affect plant performance by enhancement of nutrient acquisition, the synthesis of phytohormones, increasing tolerance and resistance to abiotic and biotic stress (Mandyam and Jumpponen 2005, Tellenbach et al. 2013). The well-known *Phialocephala fortinii* s.l.–*Acephala applanata* species complex is the dominant fungal root endophytes of coniferous trees (Grünig et al. 2002, 2004, 2006, 2008, 2011, Stenström et al. 2014, Terhonen et al. 2014). Although the DSE *P. sphaeroides* promoted the growth of Norway spruce (Terhonen et al. 2016), little is known about the molecular mechanisms underlying the observed growth promotion.

Plants including forest trees have diverse defense strategies in response to pathogen and endophyte infection. Phenolic metabolites play critical roles in defense response of conifer trees to pathogens (Witzell and Martín 2008), with activation of phenylpropanoid and flavonoid biosynthesis pathways. A proportion of the intermediate *p*-coumaroyl-CoA is allocated to the downstream of flavonoid pathway with a series of reactions including condensation, isomerization, oxidation and reduction for monomeric flavan-3-ols and polymerized proanthocyanidins (PAs) (Saito et al. 2013). Apart from phenolic metabolism, hormone signal pathways play a critical role in plant defense response. Biotrophic and necrotrophic pathogens have a contrasting nutrient strategy to exploit their host plant substrates, resulting in effective defense dependent on distinct signaling pathways. The effective defense against biotrophs is largely due to hypersensitive response (HR) with the activation of a salicylic acid (SA)-dependent signaling pathway that leads to expression of pathogenesis-related (PR) proteins. Plant defense response to necrotrophs generally depends on the activation of jasmonic acid (JA)/ethylene (ET) signaling pathways (Glazebrook 2005, Pieterse et al. 2012). The JA/ET-signaling pathway probably plays a central role in defense response in Norway spruce to the necrotrophic pathogen *H. parviporum* (Arnerup et al. 2013), whereas the SA-signaling pathway could still have an important role in Norway spruce–*Heterobasidion* interactions as the SA-mediated HR could facilitate necrotrophic pathogen infection (Likar and Regvar 2008). However, defense response of coniferous trees to endophyte colonization is unclear and has been very little studied. The effects of individual DSE colonization and the coinfection with *Heterobasidion* fungi and DSE colonization on defense response of host plants are also rarely investigated.

Our hypothesis is that a pre-inoculation of Norway spruce seedlings with a dark septate root endophyte (*P. sphaeroides)* will have some fitness benefits as well as protect the plant against infection by conifer pathogen (*H. parviporum*). To investigate the impact of the coinfection on plant defense responses, we compared the expression profiles of infected seedlings based on RNA sequencing. The results revealed some of the differences in host metabolic pathways, which are primarily involved in phenylpropanoid and flavonoid biosynthesis as well as hormone signaling that are due to the pathogen infection, the endophyte inoculation or coinfection.

## Materials and methods

### Endophyte, pathogen and plant materials

The DSE *P. sphaeroides* strain 222 was isolated from roots of Norway spruce (Terhonen et al. 2014). Homokaryotic *H. parviporum* 96026 HAMBI 2359 was provided by courtesy of Kari Korhonen (Natural Resources Institute Finland, LUKE). Norway spruce (*P. abies*) seeds (batch number: R01-98-0626-E461) were also kindly provided by LUKE. Both the endophyte and the pathogen isolates were maintained on malt extract agar (MEA: malt extract 20 g l^−1^, agar 15 g l^−1^, pH = 5.7) at 22 °C. Norway spruce seeds were surface sterilized with 30% H_2_O_2_ for 15 min, rinsed several times with sterile water and stratified for 3–4 days in the dark at 4 °C. A row of seeds was laid on the middle of a Petri dish containing 1% water agar and covered with moist, sterile filter paper. The Petri dishes containing seeds were sealed with parafilm, placed in a growth chamber for germination under a photoperiod of 16 h at 20 °C for 2 weeks.

### Dual cultures on peat-based growth media and malt extract media

Paired interactions of *H. parviporum* and *P. sphaeroides* were set up both on the peat-based growth media provided by Kekkilä Professional (Vantaa, Finland) and 1.5% malt extract media (MEA) to investigate the effect of the endophyte on the pathogen growth. The moist peat-based substrate was autoclaved for 1 h at 121 °C twice with an interval of 48 h, followed by transfer into Petri dishes (90 mm diameter). Two agar plugs (5 mm diameter), colonized with active mycelia, were inoculated face down at a distance of 60 mm on the Petri dishes with the autoclaved peat-based substrate or MEA. *H. parviporum* was added 19 days after the DSE to ensure that the DSE grew well. *H. parviporum* self-pairing was used as an additional control. The fungi were grown at 20 °C in the dark.

### Seedling inoculation and sample harvest

The DSE *P. sphaeroides* was grown on MEA plate pre-covered with a piece of cellophane membrane for 1 month. The cellophane membrane (50% size of MEA plate) containing the DSE mycelium was cut and transferred to petri dish containing modified Melin-Norkrans medium (referred now as MMN medium: 0.45 mM CaCl_2_, 0.43 mM NaCl, 0.61 MM MgSO_4_ · 7H_2_O, 4.3 mM NH_4_H_2_PO_4_, 4.7 mM KH_2_PO_4_, 30.8 μM FeCl_3_ · 6H_2_O, 0.2 μm thiamine HCl, 1 mM C_6_H_12_O_6_, agar 1.5%). Two-week-old seedlings were transferred to the medium and laid on the hyphae of the DSE. The root regions were covered with moist sterile filter paper. Autoclaved cellophane membrane without the DSE hyphae served as the uninoculated control. The plates were sealed and incubated in a growth chamber with 16 h of light at 22 °C. After 1 month, the seedlings (three to four seedlings in each plate) were transferred onto the sterile peat-based substrate in rectangular plates (230 × 82 × 18 mm) (Radia Industry Co. Ltd, Japan) and left for 1 month for substrate adaptation. Five agar plugs (5-mm-diameter) from a 17-day-old culture of *H. parviporum* were used to inoculate the seedling roots in the rectangular plate. The experimental design had four combinations (Table 1), including control seedlings without any inoculum (Pa), endophyte-infected seedlings (PaPs), pathogen-infected seedlings (PaHp) or coinfected seedlings with pathogen and endophyte (PaPsHp). Each combination had three replicates and each replicate consisted of three to four seedlings. The seedlings were grown for 1 month after pathogen inoculation before sampling and were watered every second week with 15 ml sterile water.

**Table 1:** Experimental design of Norway spruce seedlings with fungal infection.

Sample ID	Experimental condition	Description
Pa	Non-inoculated seedlings control	Seedlings under fungus-free condition
PaPs	Endophyte inoculation	Seedlings with only *P. sphaeroids* colonization
PaHp	Pathogen infection	Seedlings with only *H. parviporum* colonization
PaPsHp	Co-infectiom	Seedlings infected with the pathogen in the presence of the endophyte

At the end of the experiment, the number of lateral roots, the length of shoot and the length of primary root of the seedlings were counted or measured. One of the roots in each combination treatment was surface-sterilized (30 s in 97% EtOH, 2 min in 10% NaClO, rinsed four times with autoclaved water), cut into 5-mm pieces and incubated on 1% MEA to re-isolate the endophyte and the pathogen.

### RNA isolation

The control seedlings and infected seedlings were collected 1 month after *H. parviporum* inoculation. Samples harvested for RNA isolation consisted of the entire root system, hypocotyl and needle tissues. The tissues were immediately frozen in liquid N_2_ and stored at −80 °C. Total RNA was extracted by Cetyl trimethylammonium bromide method (Chang et al. 1993). Briefly, ground samples were mixed with 900 μl extraction buffer preheated at 65 °C and 9 μl 1M DL-Dithiothreitol solution incubated for 10 min at 65 °C and simultaneously centrifuged at 1000 r.p.m. The RNA-containing phase was separated from proteins and polysaccharides by adding chloroform:isoamyl alcohol (24:1) twice followed by centrifugation at 10,000*g*. The supernatant was mixed with 10 M LiCl overnight for RNA precipitation, and RNA was collected by centrifugation and washed with chilled absolute ethanol prior to dissolving into nuclease-free water. The total RNA quality was assessed by Agilent 2100 bioanalyzer.

### Library construction

Transcriptome sequencing (Paired-end, 150 bp) was carried out by Novogene. Sequencing libraries were generated using NEBNext® UltraTM RNA Library Prep Kit for Illumina® (NEB, USA). Briefly, mRNA was purified from total RNA by oligo(dT) beads and was fragmented randomly in fragmentation buffer, followed by cDNA synthesis using random hexamers and reverse transcriptase. After a series of procedures including terminal-repair, A-tailing, ligation of sequencing adapters, size selection and PCR amplification, PCR products were purified (AMPure XP system) and library concentration was first quantified using Qubit 2.0 fluorometer (Life Technologies). Insert size was checked on Agilent Bioanalyzer 2100 system. The library preparations were sequenced on Illumina NovaSeq 6000.

### Differential gene expression analysis

Raw reads were preprocessed by Novogene. Clean reads were obtained after removing reads with adapter contamination, or discarding reads when uncertain nucleotides constituted >10% of the reads (N > 10%) or discarding reads when low-quality nucleotides (base quality <20) constituted >50% of the reads. The quality of processed sequence data was assessed using FastQC (http://www.bioinformatics.babraham.ac.uk/projects/fastqc/). Reference genome and gene model annotation files were downloaded from the genome website (ftp://plantgenie.org/Data/ConGenIE/Picea_abies/v1.0/). The clean reads were mapped to v1.0 of the *P. abies* genome (Nystedt et al. 2013) with HISAT2. Novel gene prediction was performed by the Cufflinks Reference Annotation Based Transcript assembly method. HTSeq was used to count the read numbers mapped of each gene, including known and novel genes. FPKM (Fragments Per Kilobases of transcript sequence per Million bases) of each gene was calculated based on the length of the gene and read count mapped to this gene. The FPKM-normalized read counts were used for the downstream analyses, including principal component analysis (PCA) and differential gene expression. Differential expression analysis between two conditions was performed using DESeq2 R package. The resulting *P*-values were adjusted using Benjamini and Hochberg’s approach for controlling the false discovery rate (FDR). An FDR adjusted *P*-value of 0.05 and |log2(fold change)| of 1 were set as the threshold for significantly differential expression.

**Figure 1.**
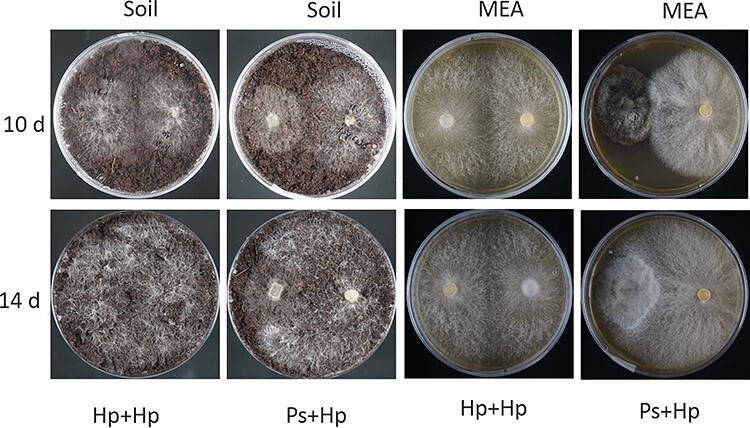
Dual cultures of *H. parviporum* (Hp: right-hand side) and *P. sphaeroides* (Ps: left-hand side) on peat-based growing media (soil) and MEA. *Phialocephala sphaeroides* had grown for 19 days prior to the inoculation of *H. parviporum*. Photos were taken 10 days and 14 days after *H. parviporum* colonization.

### Gene ontology (GO) term and Kyoto encyclopedia of genes and genomes (KEGG) pathway enrichment analysis

Biological significance of differentially expressed genes (DEGs) was explored by GO term enrichment analysis including biological process (BP), molecular function (MF) and cellular component (CC), based on the enrichment analysis tools in ConGenIE.org platform (http://congenie.org/) using all genes in Norway spruce (*Picea abies* v.1.0) as the background. Differentially expressed genes were used for further analysis of related pathways using KOBAS 3.0 (KOBAS, KEGG Orthology Based Annotation System, http://kobas.cbi.pku.edu.cn/) (Wu et al. 2006, Xie et al. 2011). The threshold of FDR-adjust *P*-value < 0.05 and the minimum number of genes for the corresponding term > 10 were considered to have statistical significance and to achieve significant enrichment.

## Results

### No antagonistic effect of the DSE on the growth of H. parviporum

In order to investigate the impact of the endophyte on the pathogen growth, dual cultures were set up in a Petri dish containing MEA or peat-based growing media. *H. parviporum*-colonized agar plug was put on media pre-colonized for 19 days by *P. sphaeroides*. Ten days later, we observed that fungal hyphae from both fungi were in contact. *H. parviporum* grew well in the presence of the fungal endophyte on both media (Figure 1). Over time, some overgrowth of the endophyte by the pathogen was observed on MEA and on peat-based growth media. No direct inhibitory effect of DSE on the homokaryotic *H. parviporum* was observed.

### The endophyte promoted root growth of Norway spruce seedlings

**Figure 2.**
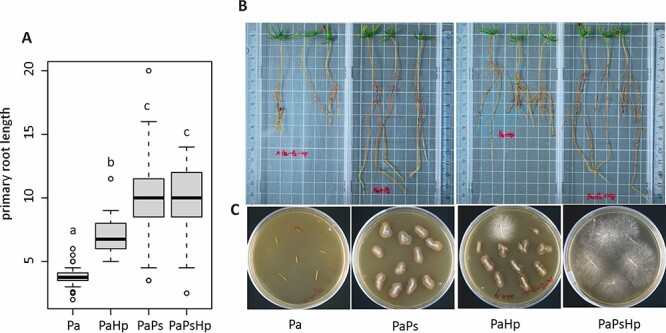
The growth of Norway spruce seedlings with fungal infection. (A) The primary root length of seedlings. (B) Effect of fungal infection on the growth of Norway spruce seedlings. (C) Fungal isolation from the infected seedlings. Pa: non-inoculated seedlings control; PaPs: *P. sphaeroides*-inoculated seedlings; PaHp: *H. parviporum*-infected seedlings; PaPsHp: *H. parviporum*-infected seedlings in the presence of *P. sphaeroides* (coinfection).

The results showed that the DSE *P. sphaeroides* promoted the root growth of Norway spruce seedlings. The length of the primary root was markedly induced in the endophyte-infected seedlings and coinfected seedlings, compared with the control seedlings and the pathogen-infected seedlings (Figure 2A and B). The DSE and pathogen successfully colonized the seedling roots, as evidenced by reisolation (Figure 2C). The endophyte-infected seedlings and coinfected seedlings had no significant difference in the number of lateral roots compared with the control seedlings (Figure S1A available as Supplementary data at *Tree Physiology* Online). The difference in the shoot length was not significant among the treatments (Figure S1B available as Supplementary data at *Tree Physiology* Online).

**Table 2:** RNA sequencing and mapping results to *P. abies* genome for each replicate. Norway spruce seedlings inoculated with *P. sphaeroides* (PaPs), *H. parviporum* (PaHp) or both of them (PaPsHp).

	Replicate	Raw reads	Clean reads	Q20 (%)	Mapped reads	Unique mapping	Multiple mapping	Exonic	Intergenic	Intronic
Pa	1	88,146,984	86,784,632	97.91	72,678,079 (83.75%)	69,940,436 (80.59%)	2,737,643 (3.15%)	66.91	29.93	3.16
	2	92,565,492	90,745,944	97.88	76,121,391 (83.88%)	73,227,495 (80.70%)	2,893,896 (3.19%)	66.77	29.85	3.38
	3	95,006,266	93,062,792	97.95	78,416,288 (84.26%)	75,476,352 (81.10%)	2,939,936 (3.16%)	67.21	29.53	3.26
PaPs	1	107,888,536	106,586,064	97.75	86,542,668 (81.20%)	83,232,031 (78.09%)	3,310,637 (3.11%)	66.65	29.65	3.69
	2	97,400,066	95,335,302	97.84	77,390,292 (81.18%)	74,447,021 (78.09%)	2,943,271 (3.09%)	66.64	29.7	3.67
	3	96,927,308	95,564,988	97.6	77,070,124 (80.65%)	74,199,805 (77.64%)	2,870,319 (3.00%)	67.55	29.18	3.28
PaHp	1	95,387,946	94,171,572	97.64	76,746,153 (81.50%)	73,851,720 (78.42%)	2,894,433 (3.07%)	66.26	30.06	3.68
	2	100,361,280	99,310,102	97.88	81,307,657 (81.87%)	78,175,863 (78.72%)	3,131,794 (3.15%)	67.37	29.36	3.27
	3	118,291,966	117,224,762	97.81	96,421,593 (82.25%)	92,776,157 (79.14%)	3,645,436 (3.11%)	65.97	30.21	3.83
PaPsHp	1	92,332,808	91,108,782	98.09	74,517,263 (81.79%)	71,689,012 (78.69%)	2,828,251 (3.10%)	66.24	30.14	3.62
	2	103,201,948	100,978,672	97.87	82,666,827 (81.87%)	79,538,227 (78.77%)	3,128,600 (3.10%)	65.73	30.6	3.67
	3	81,501,176	80,243,074	97.83	66,472,779 (82.84%)	63,952,749 (79.70%)	2,520,030 (3.14%)	66.26	30.35	3.38

### RNA sequencing and mapping of reads to Norway spruce genome

Transcriptome analysis of RNA sequence data from the four groups (control seedlings without any inoculum (Pa), endophyte-infected seedlings (PaPs), pathogen-infected seedlings (PaHp) or coinfected seedlings with pathogen and endophyte (PaPsHp)) was conducted. Twelve RNA libraries were constructed with three biological replicates for each of the four groups (Table 2). An average of 80–117 million clean reads of 150 nucleotides was obtained from each library after removal of reads with adaptor contamination, low-quality reads and reads with >10% ambiguous N bases, with an average of 97.8% of clean reads passed the quality control (Q20) (Table 2). An average of 82% clean reads was mapped to Norway spruce genome (mapped reads). Moreover, RNA sequence clean data were mapped to the genome of a closed relative *Phialocephala scopiformis* (Walker et al. 2016) and the genome of *H. parviporum* (Zeng et al. 2018). The proportion of reads mapped to *P. scopiformis* in the endophyte-infected seedlings (PaPs) and the coinfected seedlings (PaPsHp) accounted for 0.17 and 0.11% of the total sequences, respectively. The reads mapped to *H. parviporum* in PaPsHp (0.16%) was markedly lower than the reads in PaHp (1.93%).

### Comparative transcriptome response to the fungal endophyte and fungal pathogen inoculation

**Figure 3.**
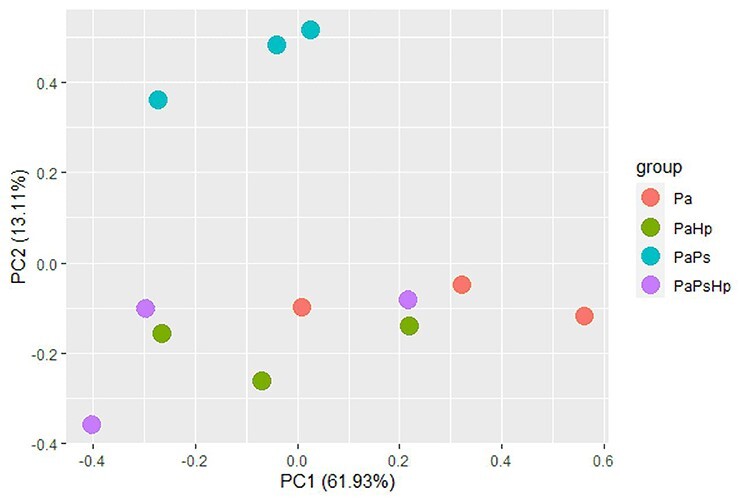
Principal component analysis based on reads counts normalized by FRKM value. Pa: non-inoculated seedlings control; PaPs: *P. sphaeroides*-inoculated seedlings; PaHp: *H. parviporum*-infected seedlings; PaPsHp: *H. parviporum*-infected seedlings in the presence of *P. sphaeroides* (coinfection).

**Figure 4.**
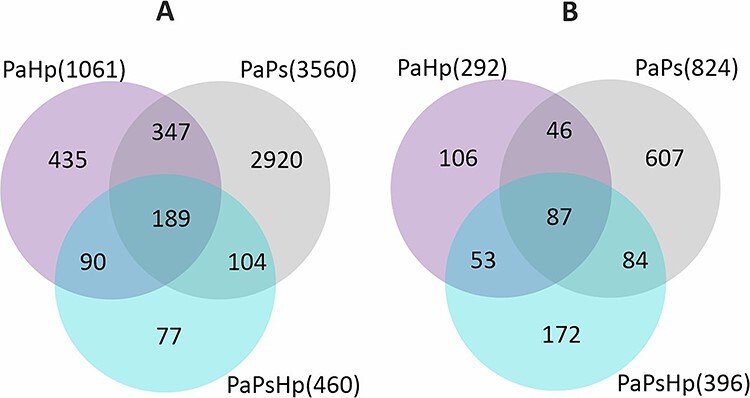
The number of genes significantly upregulated (A) and downregulated (B) during *H. parviporum* infection (PaHp), *P. sphaeroides* inoculation (PaPs) and coinfection (PaPsHp) relative to non-inoculated seedlings control (Pa).

To reveal the changes in seedlings transcriptome in response to the *P. sphaeroides* inoculation and *H. parviporum* infection, the different gene expression patterns observed using DESeq2 presented as FPKM values were compared. Principal component analysis based on the read counts normalized by FPKM showed separation of the samples of PaPs from the other conditions (Pa, PaHp, PaPsHp) (Figure 3). A total of 5269 DEGs were identified from endophyte-infected seedlings (PaPs), pathogen-infected seedlings (PaHp) and coinfected seedlings (PaPsHp) relative to the control seedlings (Pa), with a threshold of |log2(fold change)| ≥ 1 (*P*-adjust value <0.05) (Table S1 available as Supplementary data at *Tree Physiology* Online). Out of this number, 4384 DEGs were identified in PaPs (3560 upregulated, 824 downregulated), which was greater than the number of DEGs in PaHp (1061 upregulated, 292 downregulated) and PaPsHp (460 upregulated, 396 downregulated) (Figure 4). The top 20 DEGs with upregulation and downregulation levels in infected seedlings are shown in Tables S2–S7 available as Supplementary data at *Tree Physiology* Online. In PaHp, peroxidase-like genes (POX) and ethylene-responsive transcription factor (ERF) were upregulated, whereas genes related to disease resistance—cytochrome P450, auxin-responsive family were downregulated. In PaPs, 12 out of 20 genes related to ERFs were upregulated, whereas genes related to disease resistance; auxin-responsive proteins, calcium-dependent kinase and UDP-glycosyltransferases—were downregulated. In PaPsHp, the upregulated genes included transporters, transferases, lipoxygenases and ERFs. The downregulated genes encoded abscisic acid (ABA)-responsive proteins, disease resistance, auxin-responsive proteins and cytochrome P450.

Seven groups of interesting DEGs were further identified (Figure 4), consisting of a commonly shared transcriptome, three co-expression transcriptomes and three condition-specific transcriptomes. The common transcriptome was made of 276 genes simultaneously induced (189) or repressed (87) in all three conditions (Tables S8 and S9 available as Supplementary data at *Tree Physiology* Online). Co-expression transcriptomes for PaHp and PaPs, PaPs and PaPsHp, PaHp and PaPsHp are shown in Tables S10–S15 available as Supplementary data at *Tree Physiology* Online. Three groups were condition-specific transcriptomes, including PaHp-specific genes which were uniquely induced (435) or repressed (106) in PaHp, PaPs-specific induced genes (2920) or repressed (607) in PaPs, and PaPsHp-specific induced genes (77) or repressed (172) in PaPsHp (Tables S16–S21 available as Supplementary data at *Tree Physiology* Online).

### Functional enrichment analysis of the DEGs

#### GO enrichment analysis of DEGs

To evaluate the effect of fungal infection on the potential BPs, MFs and CCs of seedlings, DEGs in each group with condition-specific transcriptome, or co-expression transcriptome or common transcriptome were initially classified based on GO term enrichment analysis.

**Figure 5.**
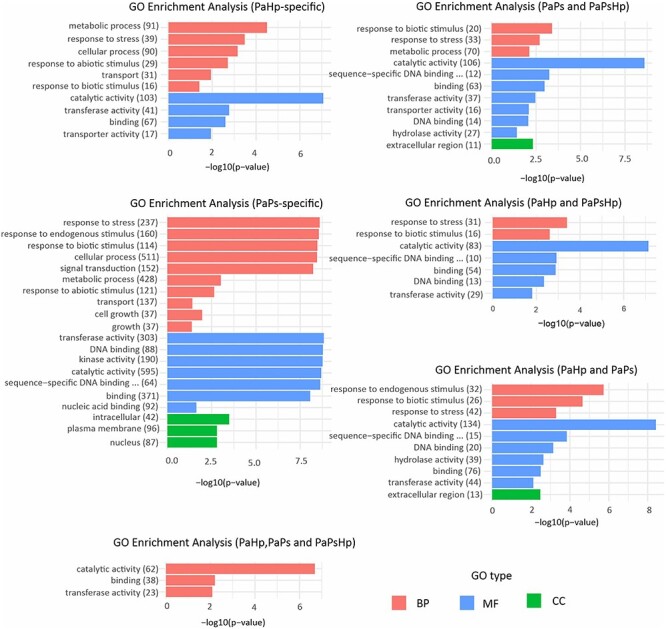
GO enrichment analysis of six groups of DEGs in seedlings during fungal infection. Two groups were condition-specific transcriptomes, including PaHp-specific genes which uniquely induced or repressed in PaHp, PaPs-specific genes only induced or repressed in PaPs, but without PaPsHp-specific genes due to the limited number of DEGs. Three groups were co-expression transcriptomes in PaPs and PaPsHp, PaHp and PaPsHp, PaHp and PaPs. One group was the common transcriptome in all three conditions (PaHp, PaPs and PaPsHp).

In condition-specific DEGs, the BP significantly enriched in PaHp and PaPs seedlings were metabolic process, response to stress, cellular process and transport. It is of particular significance that BP of DEGs in PaPs was related to signal transduction and cell growth. Molecular function GO terms in PaHp and PaPs seedlings were significant in transferase activity, catalytic activity and binding. In PaPsHp-specific samples, GO:0003824 related to catalytic activity was the only significant GO term. In co-expression transcriptome, GO terms were significant in response to stress, metabolic process, catalytic activity, transferase activity, sequence-sequence DNA binding transcription factor activity and DNA binding. Cellular component GO term was significantly related to the extracellular region. The significant GO terms of the transcriptome which were regulated in the three conditions were related to catalytic and transferase activity, and binding (Figure 5).

#### KEGG pathway enrichment analysis

According to the results of KEGG pathway enrichment analysis, DEGs in each group were classified into various biological pathways. For condition-specific transcripts (Figure S2 available as Supplementary data at *Tree Physiology* Online), a total of 541 PaHp-specific DEGs were significantly enriched in protein processing, 22 for endoplasmic reticulum, 16 for phenylpropanoid biosynthesis and 9 for flavonoid biosynthesis. PaPs-specific DEGs (3527) were also enriched in genes involved in phenylpropanoid biosynthesis (50), as well as in other major pathways including plant–microbe interaction (74), mitogen-activated protein kinases (MAPKs) signaling pathway (52) and plant hormone signal transduction (75). For PaPsHp-specific DEGs (249), only a few transcripts were associated with phenylpropanoid biosynthesis (7) and flavonoid biosynthesis (3). In terms of co-expression transcripts and common transcripts (Figure S3 available as Supplementary data at *Tree Physiology* Online), phenylpropanoid biosynthesis, linoleic acid metabolism and flavonoid biosynthesis were documented in host response to endophyte inoculation, pathogen infection and coinfection.

### Phenylpropanoid biosynthesis pathway

**Figure 6.**
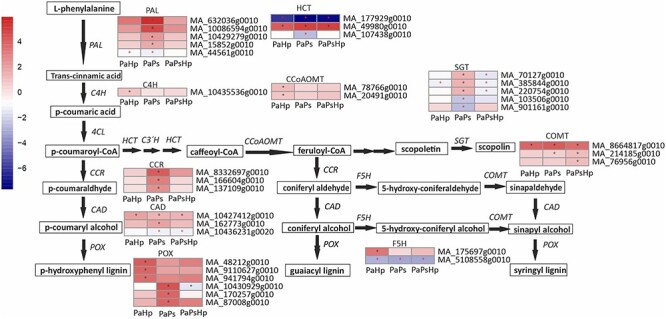
Scheme of phenylpropanoid biosynthesis pathway and the expression level of DEGs in seedlings with pathogen infection (PaHp), endophyte inoculation (PaPs) and coinfection (PaPsHp) relative to non-inoculated seedlings control. The expression level was measure by log2(fold change). Fold change is the average ratio of FPKM of genes of infected seedlings (PaPs, PaHp, PaPsHp) to FPKM of genes of non-inoculated seedlings control. FDR adjusted *P*-values <0.05 are labeled with an asterisk.

Among the enriched KEGG pathways, the pathways of phenylpropanoid biosynthesis were highly enriched in response to individual infections and coinfection. These findings suggest that genes involved in phenylpropanoid pathway played critical roles in host response to the fungal infection (Figure 6 and Figure S4 available as Supplementary data at *Tree Physiology* Online). The identified phenylpropanoid-related genes were those encoding phenylalanine ammonia lyase (PAL), *cinnamic acid 4-hydroxylase* (C4H), cinnamoyl-CoA reductase (CCR), hydroxycinnamoyl CoA shikimate/quinate hydroxycinnamoyltransferase (HCT), cinnamyl alcohol dehydrogenase (CAD), ferulate 5-hydroxylase (F5H), caffeic acid/5-hydroxyferulic acid O-methyltransferase (COMT), caffeoyl CoA 3-O-methyltransferase (CCoAOMT), peroxidase (POX) and sinapate:UDP-glucose glucosyltransferase (SGT). Genes uniquely upregulated by pathogen infection were those encoding C4H, CCoAOMT, F5H, and a part of POX involved in lignin biosynthesis. Genes exclusively upregulated by endophyte inoculation were those encoding PAL, CCR and another part of POX. The expression pattern of these phenylpropanoid-related genes showed that the expression trends of phenylpropanoid-related genes differed from *P. sphaeroides* inoculation, *H. parviporum* infection and coinfection (Figure 6).

### Flavonoid biosynthesis pathway

**Figure 7.**
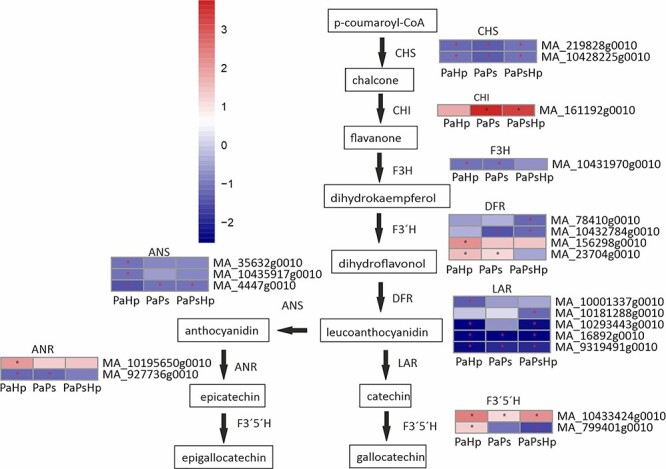
Scheme of flavonoid biosynthesis pathway and the expression level of DEGs in seedlings with pathogen infection (PaHp), endophyte inoculation (PaPs) and coinfection (PaPsHp) relative to non-inoculated seedlings control. The expression level was measure by log2(fold change). Fold change is the average ratio of FPKM of genes of infected seedlings (PaPs, PaHp, PaPsHp) to FPKM of genes of non-inoculated seedlings control. FDR adjusted *P*-values <0.05 are labeled with an asterisk.

The expression of genes encoding enzymes involved in the production of several metabolites within flavonoid biosynthesis pathway, such as monomeric flavan-3-ols and the PAs, was mostly downregulated in *H. parviporum*-infected seedlings (PaHp), *P. sphaeroides*-inoculated (PaPs) and coinfected plants (PaPsHp) (Figure 7 and Figure S5 available as Supplementary data at *Tree Physiology* Online). Two genes encoding putative chalcone synthase (CHS), which are the first steps in the flavonoid biosynthesis, were found to be downregulated in the infected seedlings. However, one putative chalcone isomerase (CHI) for the formation of flavanone from chalcone was upregulated, particularly in PaPs and PaPsHp. Additionally, one gene encoding flavanone-3-hydroxylase (F3H) was also downregulated. General downregulation of three genes encoding anthocyanidin synthase (ANS) for the formation of anthocyanidin from leucoanthocyanidin, and five genes encoding leucoanthocyanidin reductase (LAR), a branching enzyme involved in the biosynthesis of 2,3-trans-(+)-flavan-3-ols, was equally observed.

A mixed pattern of differential expression occurred for the remaining genes, including those encoding flavonoid 3′5′-hydroxylase (F3′5′H), dihydroflavonol reductase (DFR) and anthocyanidin reductase (ANR). Two genes were assigned to encode F3′5′H with one upregulated in all infected seedlings and another induced in PaHp but reduced in PaPsHp. The expression of four genes encoding putative DFR, which catalyzes the reduction of the 4-keto group of dihydroflavonol to the corresponding leucoanthocyanidin, varied in the different treatments. Two genes were downregulated in PaPsHp, one of which was also downregulated in PaPs. One gene was significantly upregulated in PaHp, and another was induced by individual infections. Induction of one gene encoding ANR was observed during fungal infection, with a higher expression level in PaHp. However, another gene encoding ANR was downregulated during fungal infection (Figure 7 and Figure S5 available as Supplementary data at *Tree Physiology* Online).

#### Plant hormone signal transduction

**Figure 8.**
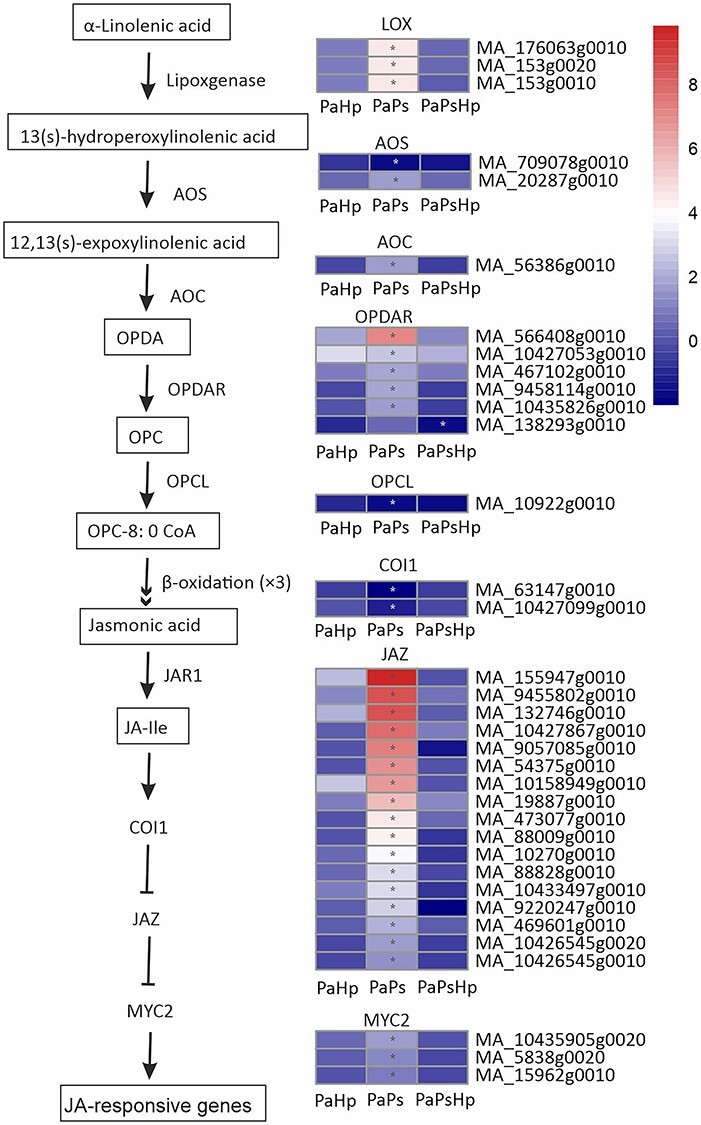
Scheme of JA biosynthesis and signaling pathway and the expression level of DEGs in seedlings with pathogen infection (PaHp), endophyte inoculation (PaPs) and coinfection (PaPsHp) relative to non-inoculated seedlings control. Fold change is the average ratio of FPKM of genes of infected seedlings (PaPs, PaHp, PaPsHp) to FPKM of genes of non-inoculated seedlings control. FDR adjusted *P* values <0.05 are labeled with an asterisk.

Jasmonic acid and related compounds are key plant hormones regulating defense response against microbial pathogens (Ruan et al. 2019). Jasmonic acids are synthesized from *α*-linolenic acid, which is converted by lipoxygenases (LOX). In our study, all of the predicted LOX-coding genes were significantly induced in PaPs. The subsequent process for the JAs formation needs allene oxide synthase (AOS), allene oxide cyclase (AOC), OPDA reductase (OPDAR) and OPCL. One gene encoding AOS was significantly downregulated, whereas another was significantly upregulated in PaPs. Five out of six predicted genes encoding OPDAR were significantly induced, and one OPCL-coding gene was significantly repressed in PaPs. JAZ, generally regarded as a repressor in the JA signaling pathway and was targeted by COI1 for degradation to relieve the downstream gene expression (Ruan et al. 2019). Two COI1-coding genes were significantly downregulated and all of the predicted JAZ-coding genes were upregulated in PaPs. MYC2-coding genes transcription factors bind with JAZ repressors and could be activated by the degradation of JAZ. In PaPs, three MYC2 genes were significantly upregulated (Figure 8).

**Figure 9.**
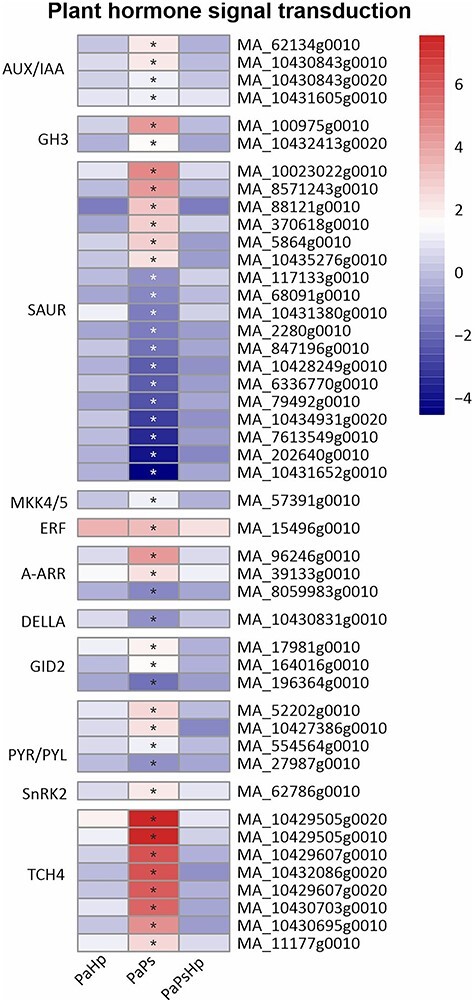
Heatmap of the expression level of genes related to plant hormone signal transduction. The expression level was measured by log2(fold change). Fold change is the ratio of FPKM of genes of infected seedlings (PaPs, PaHp, PaPsHp) to FPKM of genes of non-inoculated seedlings control. FDR adjusted *P*-values <0.05 are labeled with an asterisk.

Genes related to auxin (AUX), ethylene (ET), cytokinin (CK), gibberellin (GA), abscisic acid (ABA) and brassinosteroid signaling pathway were differentially expressed in PaPs. In the AUX signaling pathway, several auxin-responsive genes encoding AUX/IAA and GH3 were significantly upregulated in PaPs. A total of 18 genes were related to auxin-responsive protein SAUR21-like (SAUR), with six genes upregulated and twelve genes downregulated. An upregulation of one gene related to ERF in the ET signaling pathway was observed. In the CK signaling pathway, two genes related to the two-component response regulator ARR-A family (A-ARR) were significantly induced and one A-ARR gene was reduced. In the GA signaling pathway, one gene associated with DELLA proteins, which function as growth suppressors, was weakly downregulated. Two out of three predicted F-box protein GID2 were upregulated in PaPs, whereas the rest GID2-coding genes were downregulated. A total of five genes involved in the ABA catabolism showed increased transcript levels, including abscisic acid receptor PYR/PYL family (PYR/PYL) and serine/threonine-protein kinase (SRK2), except for PYR/PYL gene, which was downregulated. In the brassinosteroid signaling pathway, a total of eight genes encoding xyloglucan: xyloglucosyl transferase TCH4 (TCH4) were significantly induced in PaPs (Figure 9).

**Figure 10.**
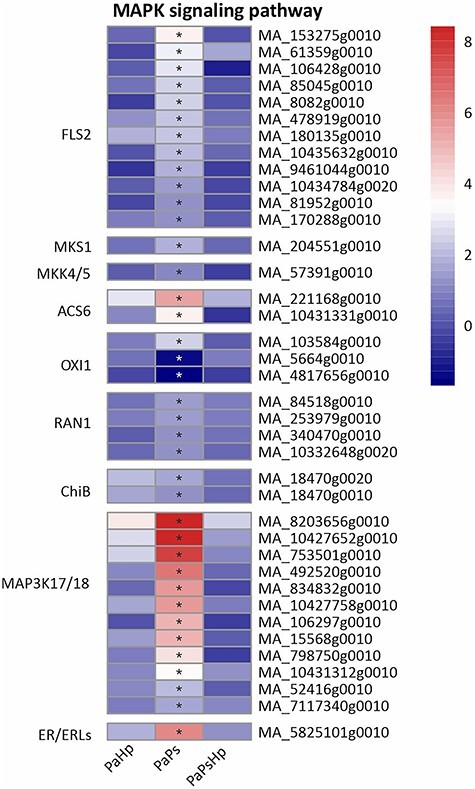
Heatmap of the expression level of genes related to MAPK signaling pathway. The expression level was measured by log2(fold change). Fold change is the ratio of FPKM of genes of infected seedlings (PaPs, PaHp, PaPsHp) to FPKM of genes of non-inoculated seedlings control. FDR adjusted *P*-values <0.05 are labeled with an asterisk.

Plant hormones mediate plant defense responses, which are also interconnected with sequential phosphorylation of plant MAPKs (Cristina et al. 2010). Genes related to MAPKs were specifically upregulated by endophyte inoculation (Figure 10). 1-aminocyclopropane-1-carboxylate synthase 6 (ACS6), the rate-limiting enzyme of ethylene biosynthesis, is the substrate of MPK3/6, which is activated by the active form MKK4/5 (Cristina et al. 2010, Liu and Zhang 2004). An MKK4/5-coding gene was slightly upregulated and two ACS6-coding genes were strongly induced. Four DEGs involved in P-type Cu + transporter (RAN1) and are essential for ethylene signaling were slightly upregulated. A total of 12 DEGs mainly putative MAP3K17/18, were found to be upregulated in PaPs (Figure 10).

### Calcium-mediated signaling

**Figure 11.**
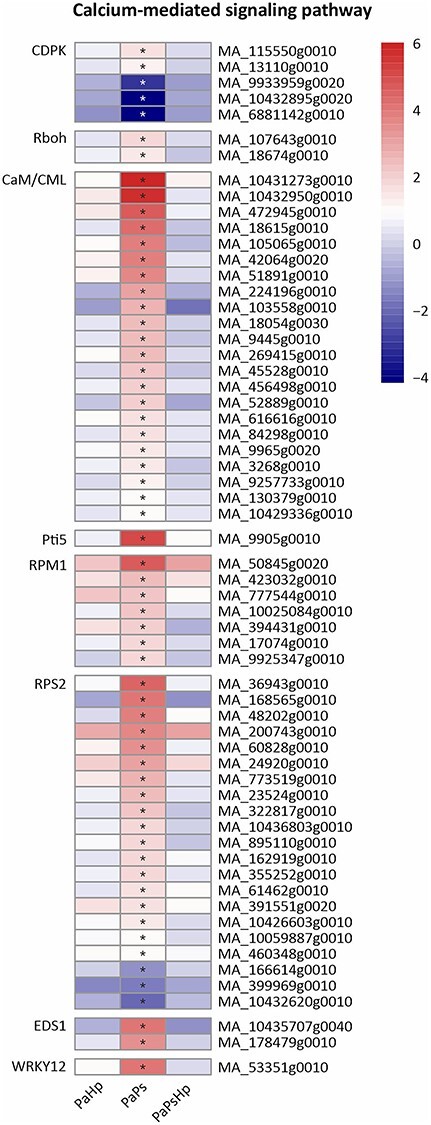
Heatmap of the expression level of genes related to Ca^2+^ signaling pathway. The expression level was measured by log2(fold change). Fold change is the ratio of FPKM of genes of infected seedlings (PaPs, PaHp, PaPsHp) to FPKM of genes of non-inoculated seedlings control. FDR adjusted *P*-values <0.05 are labeled with an asterisk.

Calcium (Ca^2+^) signaling plays a fundamental role in growth, development and stress responses in plants (Valmonte et al. 2014). Calmodulins or calmodulin-like proteins (CaM/CML), calcium-dependent protein kinases (CDPK) are two key Ca^2+^ sensors, which were differentially expressed in PaPs. A total of 22 genes encode putative CaM/CML proteins and all of them were expressed in PaPs. Two CDPK-coding genes were induced, whereas three CDPK-coding genes were reduced in PaPs. Respiratory burst oxidases (Rboh) encode the plant NADPH oxidases, which respond to various stress by an accumulation of Reactive oxygen species (ROS) (Wang et al. 2018). Two Rboh genes were significantly upregulated in PaPs. A total of 21 disease resistance proteins RPS2 were found in PaPs, of which 18 genes were upregulated and 3 of the genes downregulated. Seven disease-resistance proteins RPM1 were induced in PaPs. One WRKY12-coding gene was significantly upregulated in PaPs (Figure 11).

## Discussion

*Phialocephala sphaeroides* has been found mostly in very acid forest soil, for the first time in Canada, later in Finland and never in central Europe. In this study, *P. sphaeroides* caused a marked increase in the primary root length of Norway spruce seedlings. Plant growth-promoting DSEs are likely to facilitate nutrient uptake (Jumpponen et al. 1998, Mandyam and Jumpponen 2005, Usuki and Narisawa 2007), mineralizing organic nitrogen and phosphorus in inorganic forms for easy utilization by plants (Della Monica et al. 2015, Surono Narisawa 2017). Dark septate endophyte inoculation is able to change the root architecture of the host plants and promote root growth to absorb nutrients and water in soil (Li et al. 2019). The modulation of root architecture involves auxin pathways which are related to cell division, cell enlargement and defense response (Egamberdieva et al. 2017, Sukumar et al. 2013). Some DSE fungi including *Phialocephala* genus could produce auxin (Berthelot et al. 2016). The genome of *P. subalpina* and *P. scopiformis*, the closely related species of *P. sphaeroides*, showed that they contain numerous genes encoding phosphatases, and several genes encoding auxin-resistance proteins and auxin efflux carriers (Schlegel et al. 2016, Walker et al. 2016), which might be related to the plant growth promotion. Additionally, some conifer endophytes are mutualistic symbionts, due in part to antagonism toward plant pathogens and insects. Although *P. sphaeroides* was expected to inhibit the growth of *H. parviporum* (Terhonen et al. 2016), the strain used in this study probably lost the antagonistic ability as shown in the dual culture assay as previously reported (Wen et al. 2019). Some *P. subalpina* isolates effectively reduced mortality and disease intensity caused by root pathogens (Tellenbach and Sieber 2012). Sclerin and sclerotinin A produced by the root endophyte *Phialocephala europaea* were found to be toxic to the root dieback pathogen *Phytophthora plurivora* (Tellenbach et al. 2013). Conifer foliar endophytes also produce antifungal or anti-insect compounds, such as griseofulvin by *Xylaria* sp., pyrenophorol by *Lophodermium* sp., vermiculine by *Phialocephala* sp. and rugulosin by *P. scopiformis* (Frasz et al. 2014, McMullin et al. 2018, Richardson et al. 2014). Rugulosin has been reported to effectively reduce the growth of spruce budworm larvae (Miller et al. 2002, 2008). It is likely that the colonization level of *P. sphaeroides* as documented by RNA-seq data was not significantly affected by the presence of the pathogen. On the other hand, the colonization level of the pathogen as also shown by RNA-seq data was significantly limited by the presence of the root endophyte. This limitation could probably be a result of secondary metabolites produced by the root endophyte in vivo.

RNA-seq analysis of infected seedlings and control seedlings showed that the number of DEGs was highest in *P. sphaeroides*-inoculated seedlings. This suggested a major positive influence on host transcriptional regulation by the endophyte. By contrast, the number of DEGs was reduced in coinfection. The result probably indicates that the subsequent pathogen (*H. parviporum*) infection triggered Norway spruce reprogramming of host metabolisms in dual inoculation with the fungal root endophyte (*P. sphaeroides*) without negative impact on the plant health. The specific or common transcriptome indicated a specific or general spruce response to endophyte inoculation, pathogen infection or coinfection, with genes mainly enriched in phenylpropanoid and flavonoid biosynthesis, JA biosynthesis, MAPK signaling pathway, plant hormone signal transduction and plant–microbe interaction.

The phenylpropanoid biosynthesis pathway was generally activated during fungal infection, but the gene expression patterns varied between treatments. *Heterobasidion* infection-induced genes Which are involved in the phenylpropanoid pathway, corresponding to the accumulation of phenolics and cell wall thickening as a result of lignification (Adomas et al. 2007, Oliva et al. 2015). Other authors have reported high levels of piceasides and flavonoids in the less susceptible genotype of Norway spruce in response to *Heterobasidion* infection (Danielsson et al. 2011). Four genes involved in the phenylpropanoid and flavonoid pathways (HCT, 4CL, MYB, LAR) influenced resistance traits against *H. parviporum* in *P. abies* (Lind et al. 2014). Genes related to PAL and POX showed a spatial and temporal expression pattern in response to *Heterobasidion* infection (Deflorio et al. 2011, Likar and Regvar 2008). Four PAL-coding genes were found to be significantly induced after the DSE inoculation alone, whereas their expression levels in the coinfected seedlings were not significant but comparable to the observations with the pathogen. These results suggested that the subsequent infection with *H. parviporum* was able to influence the phenylpropanoid metabolism modulated by *P. sphaeroides*. *Heterobasidion* infection was also reported to affect ectomycorrhizal symbiosis process (Zampieri et al. 2017). Class III peroxidases exist as a multigene family in Arabidopsis (Tognolli et al. 2002), and a variety of them also contributed to lignin polymerization in Norway spruce (Koutaniemi et al. 2007, Warinowski et al. 2016). An overlapping set of POX-coding genes was expressed during fungal infection, whereas a set of POX-coding genes was specifically upregulated by the pathogen or by the endophyte.

Some key genes in flavonoid biosynthesis were found to be downregulated. LAR and flavonoid 3′,5′-hydroxylase (F3′5′H) played important roles in accumulation of catechin and gallocatechin in Norway spruce (Hammerbacher et al. 2014, 2018). PaLAR3-coding has been reported to influence the resistance of *Picea abies* against *H. parviporum* (Nemesio-Gorriz et al. 2016). However, *LAR*, as well as certain other important host defense genes (*CHS, F3H, ANS*), showed decreased transcript levels in fungal inoculated roots in this study. The downregulation of genes in the flavonoid biosynthesis was also found in blueberry inoculated with a plant growth-promoting DSE (Wu et al. 2020). The fungal endophyte *Epichloë festucae* increased ryegrass resistance to fungal pathogens, whereas it also reduced plant growth. The authors found that the host phenylpropanoid and flavonoid biosynthetic pathways were activated at a cost to primary metabolism and photosynthesis (Dupont et al. 2015). The upregulation of genes involved in lignin biosynthesis and the general downregulation of genes in flavonoid biosynthesis might indicate the increase in the flux of phenylalanine into lignin biosynthesis and decrease into flavan-3-ols and proanthocyanidins during fungal infection.

Plant growth promotion is regulated by the modulation of hormone levels. Some endophytic fungi and bacteria that promote plant growth cause upregulation of genes involved in auxin biosynthesis and metabolism, ethylene synthesis, gibberellin biosynthesis and cytokinin metabolism (Galambos et al. 2020, Ortiz et al. 2019). The endophyte *P. sphaeroides* modulated hormone signal transduction in Norway spruce seedlings, which partly contributed to the promotion of root growth. Multiple genes involved in JA biosynthesis and signaling pathways were also upregulated by *P. sphaeroides* alone. The JA-dependent signaling pathway was probably activated by the fungal endophyte in Norway spruce. Mitogen-activated protein kinase signaling and calcium (Ca^2+^) signaling play critical roles in transcriptional reprogramming (Tsuda and Somssich 2015). Mitogen-activated protein kinase (MAPK) cascades phosphorylate substrates including transcription factors to regulate downstream of gene expression (Meng and Zhang 2013, Zhu et al. 2016). MAP3K17 and MAP3K18 were likely regulated by ABA via ABA core signaling module composed of PYR/PYL receptors and SnRK2 (Danquah et al. 2015). Abscisic acid core signaling modules (PYR/PYL, SnRK2) and numerous MAP3K17/18 genes were induced by the endophyte inoculation alone. This might indicate that MAPK signaling is involved in ABA signal transduction in Norway spruce upon *P. sphaeroides* inoculation. CaM/CML and CDPKs perceive the fluctuations of cytosolic Ca^2+^ upon stress response, which regulates protein phosphorylation and transcriptional controls in plants (Poovaiah et al. 2013). CDPK has been proposed to phosphorylate NADPH oxidase Rboh and monitor the homeostasis of reactive oxygen species (Kobayashi et al. 2007). The upregulation of genes encoding CDPK, CaM/CML and Rboh might suggest alteration of reactive oxygen species in Norway spruce in response to *P. sphaeroides* inoculation. Jasmonic acid biosynthesis, plant hormone transduction pathway, MAP kinase cascade pathway and Ca^2+^ signaling were specifically activated by *P. sphaeroides* inoculation alone, which might collectively contribute to the promotion of seedling root growth. The subsequent *H. parviporum* infection triggered Norway spruce reprogramming of its metabolism, surprisingly without notable adverse effect on root growth.

## Conclusions

The DSE *P. sphaeroides* was able to promote root growth of Norway spruce seedlings. Transcriptome changes in *P. sphaeroides*-inoculated, *H. parviporum*-infected and coinfected seedlings of Norway spruce resulted in general metabolic responses particularly in genes involved in phenylpropanoid and flavonoid biosynthesis. The DSE inoculation alone activated specific transcriptional responses on genes responsible for JA biosynthesis, plant hormone transduction, MAPK signaling and Ca^2+^ signaling, potentially contributing to the promotion of root growth. The coinfection suppressed the induction of numerous genes without a negative impact on the growth of spruce seedlings. We concluded that the subsequent pathogen *H. parviporum* infection triggered reprogramming of host metabolism in dual inoculation with the fungal root endophyte *P. sphaeroides*. Conversely, the endophyte counteracted the adverse negative effects of the pathogen on the plant growth. It is most likely that the fungal endophyte might have a potential protective function for roots of young seedlings in forest nurseries before out-planting. Such protective function merits further investigation.

## Supplementary Material

Supplementary_Information_tpab147

Table_S1_tpab147

Table_S2-S7_tpab147

Table_S8-S15_tpab147

Table_S16-S21_tpab147

## Data and materials availability

The datasets generated and analyzed during this study are available in the NCBI SRA database (https://www.ncbi.nlm.nih.gov/sra/) under the accession number SRX11066039-SRX11066050. BioProject PRJNA734471 (https://www.ncbi.nlm.nih.gov/sra/?term=PRJNA734471) as well as its additional files (https://www.ncbi.nlm.nih.gov/Traces/study/?acc=PRJNA734471).
