# Supplementary material for: The dark septate endophyte Phialocephala sphaeroides confers growth fitness benefits and mitigates pathogenic effects of Heterobasidion on Norway spruce
Source: Tree Physiol. 2021 Nov 15;42(4):891–906. doi: 10.1093/treephys/tpab147 (PMC9000907; doi:10.1093/treephys/tpab147)
Supplement: Supplementary_Information_tpab147 [file supplementary_information_tpab147.docx]

**The dark septate root endophyte *Phialocephala sphaeroides* confers growth fitness benefits and mitigates pathogenic effects of *Heterobasidion* on Norway spruce**

Zilan Wen ^1^, Eeva Terhonen^2^, Fred O. Asiegbu ^1*^

^1^ Faculty of Agriculture and Forestry, P. O. Box 27, Latokartanonkaari 7, University of Helsinki, 00014 Helsinki, Finland; zilan.wen@helsinki.ﬁ (ZW)

^2^Natural Resources Institute Finland (Luke), Forest health and biodiversity, Latokartanonkaari 9, FI‐00790 Helsinki, Finland, E-mail: [eeva.terhonen@luke.fi](mailto:eeva.terhonen@luke.fi) (ET)

*For correspondence. E-mail: fred.asiegbu@helsinki.ﬁ (FOA); Tel.: +358-294158109

**Supplemental figures and figure legends**

**
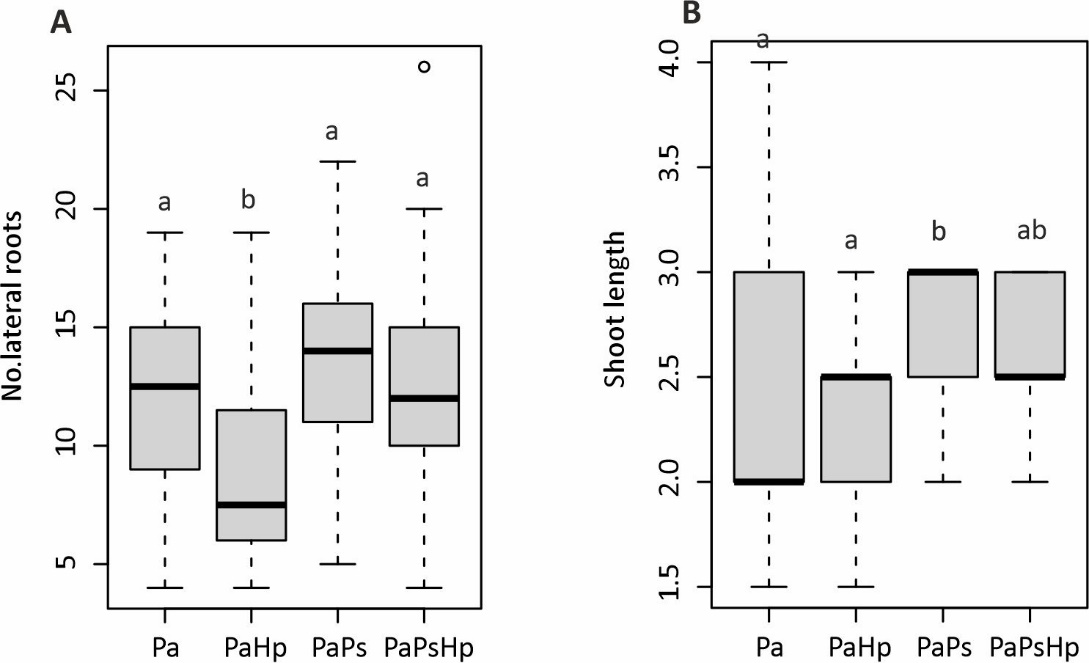
**

**Figure S1. The growth of Norway spruce during fungal infection.** (A) The number of lateral roots. (C) Shoot length. 20-30 seedlings were collected one month after *H. parviporum* infection. Different letters indicate the significant differences among treatments according to one-way ANOVA (p ≤ 0.05).


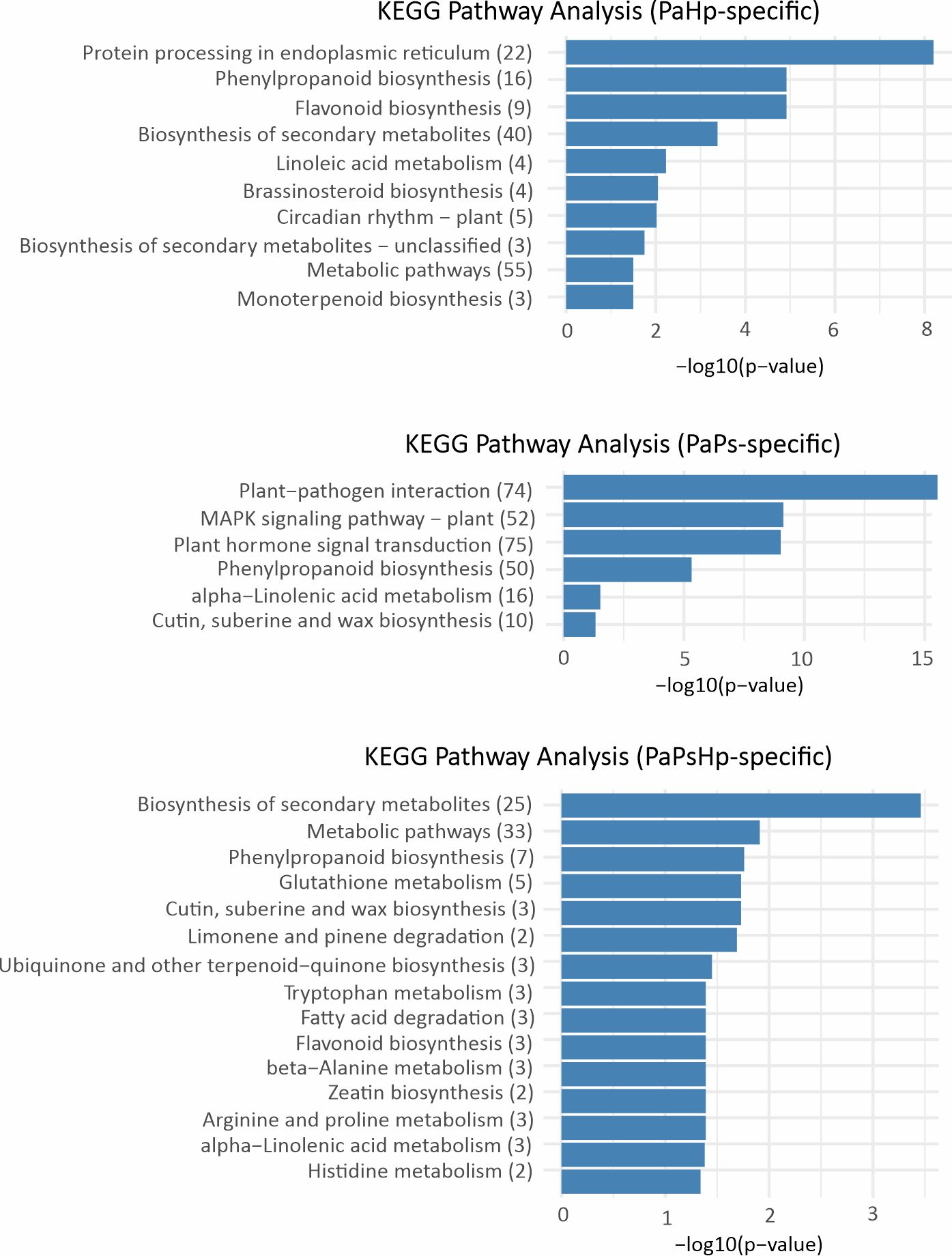


**Figure S2. KEGG enrichment pathway analysis of condition-specific DEGs in seedlings during fungal infection.**


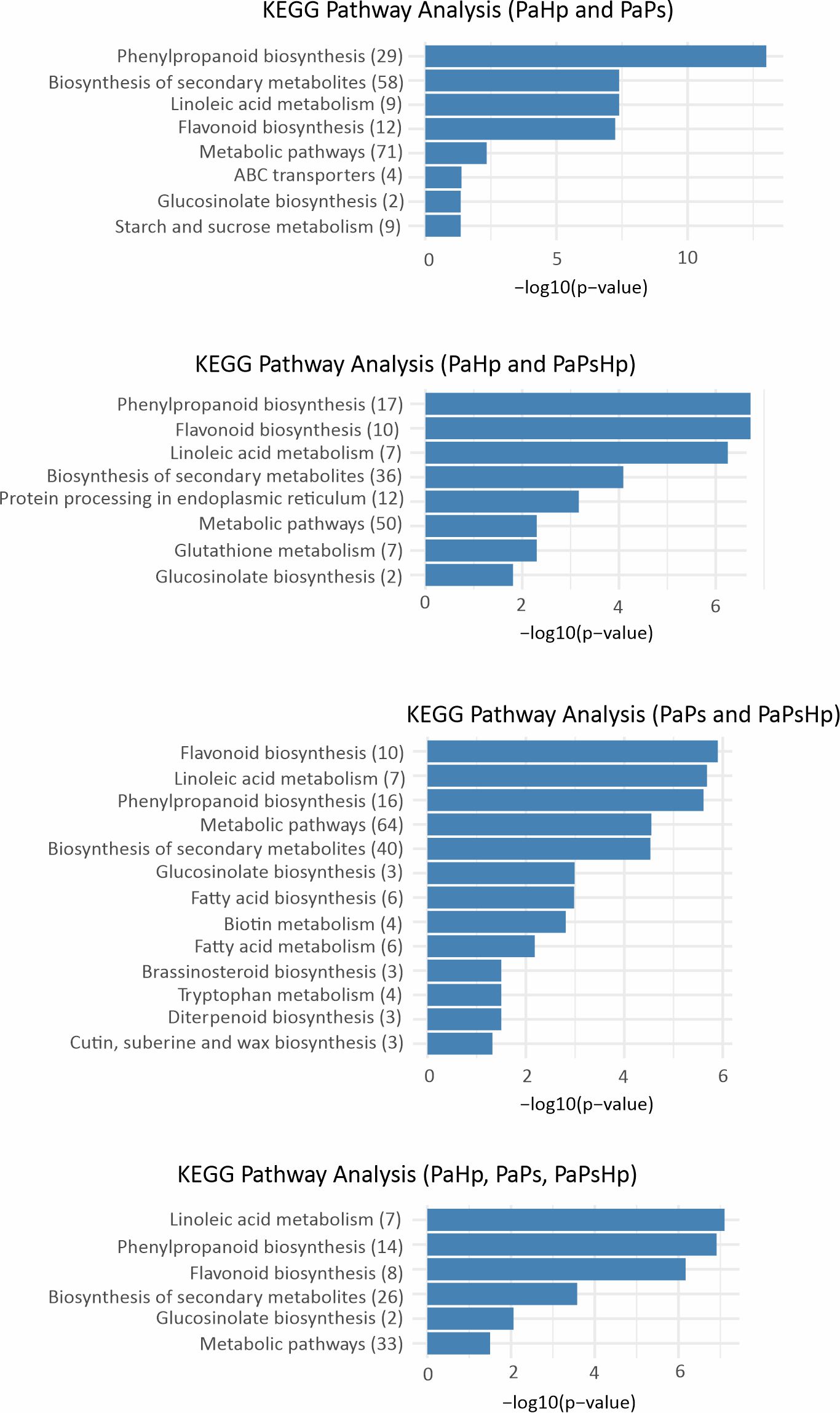


**Figure S3. KEGG enrichment pathway analysis of common DEGs in seedlings during fungal infection.**


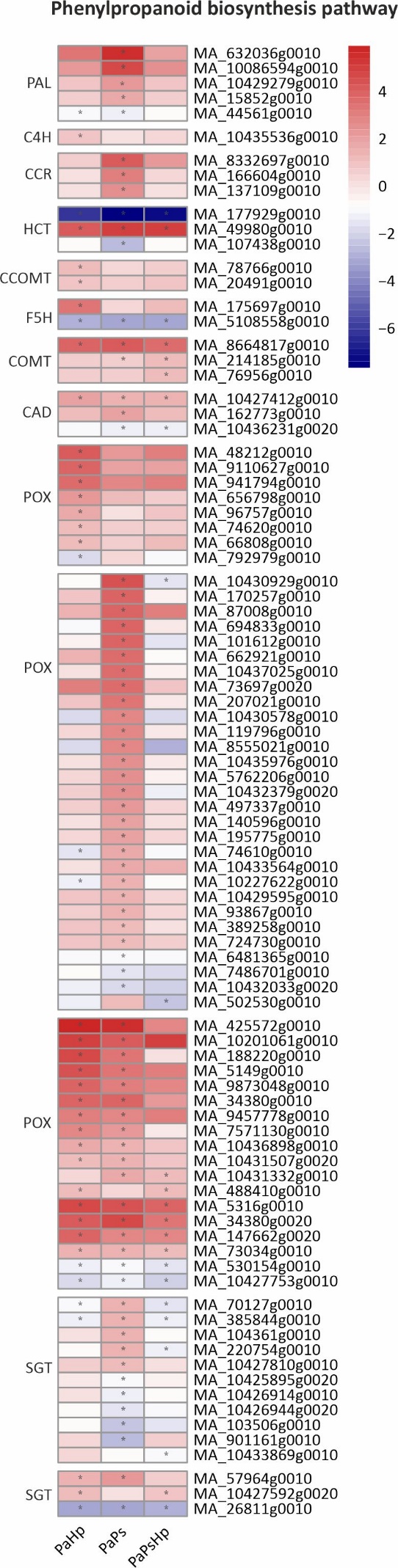


**Figure S4. Heatmap of the expression level of genes related to phenylpropanoid biosynthesis pathway.** The expression level was measure by log2(fold change). Fold change is the average ratio of FPKM of genes of infected seedlings (PaPs, PaHp, PaPsHp) to FPKM of genes of control seedlings. FDR adjusted P-values less than 0.05 were labeled as ‘*’. Phenylalanine ammonia lyase (PAL); Cinnamic acid 4-hydroxylase (C4H), Cinnamoyl-CoA reductase (CCR), Hydroxycinnamoyl CoA shikimate/quinate hudroxycinnamoyltransferase (HCT); Cinnamyl alcohol dehydrogenase (CAD); Ferulate 5-hydroxylase (F5H); Caffeic acid/5-hydroxyferulic acid O-methyltransferase (COMT); Caffeoyl CoA 3-O-methyltransferase: CCoAOMT; Peroxidase (POX); Sinapate:UDP-glucose glucosyltransferase (SGT).


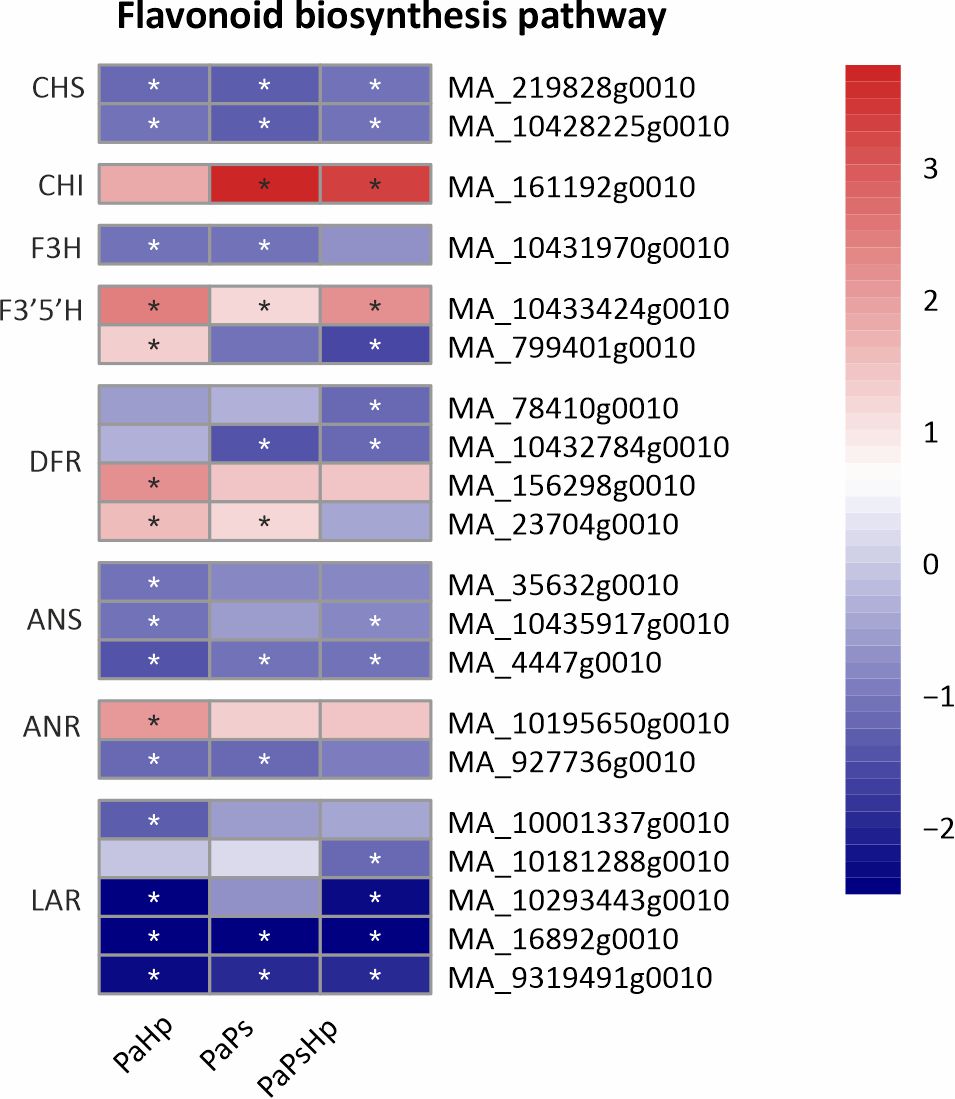


**Figure S5. Heatmap of the expression level of genes related to flavonoid biosynthesis pathway.** The expression level was measure by log2(fold change). Fold change is the average ratio of FPKM of genes of infected seedlings (PaPs, PaHp, PaPsHp) to FPKM of genes of control seedlings. FDR adjusted P-values less than 0.05 were labeled as ‘*’. Chalcone synthesis (CHS); Chalcone isomerase (CHI); Flavanone-3-hydroxylase (F3H); Flavonoid 3’5’-hydroxylase (F3’5’H); Dihydroflavonol reductase (DFR); Leucoanthocyanidin reductase (LAR); Anthocyanidin synthase (ANS); Anthocyanidin reductase (ANR).

**Supplemental table legends**

Table S1. Fragments Per Kilobases of transcript sequence per Millions base (FPKM) of differentially expressed genes (DEGs) identified in Norway spruce seedlings without inoculation (Pa), and seedlings inoculated with *P. sphaeroides* (PaPs), or *H. parviporum* (PaHp), or both of them (PaPsHp).

Table S2-S7. Top 20 differentially expressed genes (DEGs) upregulated (S2) and downregulated (S3) in *H. parviporum*-infected seedlings. Top 20 DEGs upregulated (S4) and downregulated (S5) in *P. sphaeroides*-infected seedlings. Top 20 DEGs upregulated (S6) and downregulated (S7) in seedlings with coinfection.

Table S8-S15. DEGs commonly upregulated (S8) and downregulated (S9) in seedlings with coinfection. DEGs commonly upregulated (S10) and downregulated (S11) in seedlings with pathogen infection and endophyte inoculation. DEGs commonly upregulated (S12) and downregulated (S13) in seedlings with endophyte inoculation and coinfection. DEGs commonly upregulated (S14) and downregulated (S15) in seedlings with pathogen infection and coinfection.

Table S16-S21. DEGs specifically upregulated (S16) and downregulated (S17) in seedlings with endophyte inoculation. DEGs specifically upregulated (S18) and downregulated (S19) in seedlings with pathogen infection. DEGs specifically upregulated (S20) and downregulated (S21) in seedlings with coinfection.
